# Analysis of Students' Sports Exercise Behavior and Health Education Strategy Using Visual Perception–Motion Recognition Algorithm

**DOI:** 10.3389/fpsyg.2022.829432

**Published:** 2022-05-13

**Authors:** Minwei Chen, Yunzheng Zhou

**Affiliations:** ^1^College of Physical Education, Chongqing University, Chongqing, China; ^2^College of Physical Education and Physical Medicine, Chongqing Medical University, Chongqing, China

**Keywords:** health education, vision sensing, skeleton recognition, artificial intelligence algorithm, Hidden Markov Model

## Abstract

This study aims to explore the future development path of the college health education and health education's impact on students' sports exercise. Specifically, artificial intelligence (AI) algorithm is combined with intelligent robotics technology to acquire and analyze students' sports exercise behaviors. As a result, a new development model is formulated for college health education. First, it explores students' sports exercise and health education situation in Chinese higher institutions and uncovers the underlying problems. Then it puts forward the corresponding modification suggestions. Second, the AI algorithm and the Kinect sensor-mounted intelligent robot capture the human skeleton features to obtain smooth skeleton joint points data. At the same time, a visual perception human motion recognition (HMR) algorithm is established based on the Hidden Markov Model (HMM). Afterward, the proposed HMM-based HMR algorithm is used to recognize students' sports exercise motions by analyzing human motion skeleton images. The experimental outcomes suggest that the maximum reconstruction error of the HMR algorithm is 10 mm, and the compression ratio is between 5 and 10; the HMR rate is more than 96%. Compared with similar algorithms, the proposed visual perception HMR algorithm depends less on the number of training samples. It can achieve a high recognition rate given only a relatively few samples. Therefore, the proposed (AI + intelligent robot)-enabled HMM-based HMR algorithm can effectively identify the behavior characteristics of students in sports exercise. This study can provide a reference for exploring college students' health education development path.

## Introduction

The health education is an integral part of school teaching and plays a vital role in improving students' health quality. However, statistics imply that Chinese teenagers dedicate less time to sports exercise than their foreign peers. Also noticed is that they are not allowed enough time for medium and high-intensity training critical to the healthy development of teenagers. Such issues have increased Chinese students' overweight and obesity rates (De Steenberg, 2019). Additionally, the current school health education system centers around physical education (PE) and has demonstrated many shortcomings. For example, the school seldom provides comprehensive training for PE faculty. The PE curriculum lacks innovation, normalization, and interestingness. Thus, it discourages the students from participating in extracurricular sports activities (Huang, 2019). Undeniably, advanced scientific and technological development, such as computer technology (CT), has brought humanity a more convenient and efficient life. Nevertheless, the new technologies' impact on students' health cannot be ignored. Students nowadays spend too much time on electronic equipment out of their already limited free time. No wonder students' physical quality will decline, and the obesity rate raises. Fortunately, it is feasible to use modern technological means, such as the human motion recognition (HMR) algorithm, to analyze students' motions and recognize and perceive the significance of human motions (Wang et al., 2018). In particular, artificial intelligence (AI) technology has become a research and application hotspot by helping people solve many problems through intellectualized machines and robots. Therefore, this study uses an intelligent algorithm combined with intelligent robotics technology to analyze students' sports exercise behavior and establish a scientific health education scheme. The proposed method can improve the technical scheme of school sports equipment and sports training management mode. It can scientifically guide students' sports exercise behavior and improve school health education.

Foreign and domestic scholars have carried out extensive research on HMR. Qiu et al. (2019) proposed a visual-scale image 3D tracking algorithm based on correlation analysis to track and measure dynamic objects' distances accurately. The algorithm could accurately track anything without any prior conditions about the thing (Qiu et al., 2019). Yang et al. (2019) used Kinect motion sensors to connect with mechanical axes to simulate human visual perception behaviors. They also utilized the Kalman filter to control the three-axis motion machine to perceive automatically and visually position real-time human motions. They applied it to distance education (Yang et al., 2019). Khan et al. (2019) developed a gait recognition algorithm based on spatiotemporal motion features of human gait. This algorithm could extract local spatiotemporal descriptors from gait video sequences and use vector encoding and Gaussian mixture models to encode the descriptor. Then, they classified encoded features using support vector machine (SVM) to identify the individuals. After the experiments, they concluded that the method had an excellent performance on five databases (Khan et al., 2019). Huang and Sun (2020) applied augmented reality (AR) real-time depth image technology to 3D HMR. They established a sensor-based visual inertia initialization algorithm. The proposal offered an illumination-robust solution for 3D HMR against the changes of visual features. This algorithm was integrated with two frames of image sequences to provide accurate initial values for vision-based motion estimation, thereby improving the HMR accuracy (Huang and Sun, 2020). Sun et al. (2019) used transfer learning (TL) and neural networks (NNs) to recognize the human gait and predict the subsequent motion of the human body by capturing the behavior of the task in images or videos. Through the experiments, they found that TL improved the detection performance of the algorithm (Sun et al., 2019). Many studies have provided research on HMR through AI methods and have put forward various comprehensive research algorithms. Nevertheless, the accuracy of HMR needs to be improved at this stage, which few studies have involved, and even fewer studies apply HMR in health education.

Existing studies have proved that visual HMR algorithms can recognize and capture human motions effectively. Therefore, to study the characteristics of students' motion behaviors, this study establishes an HMR algorithm by using AI and intelligent robotics technology. The intelligent robot mounted with Kinect sensors can recognize students' exercise behavior and scientifically guide students' exercise. Based on data analysis, the intelligent robot can also give corresponding improvement suggestions against the problems in school health education. This research can provide an effective HMR algorithm and a reference for studying human motion through algorithms. Therefore, this study is breakthrough research, which provides the main technical support for the comprehensive feature analysis of human motions. Meanwhile, it provides an effective method for health education and identifying students' daily sports exercises to improve classroom efficiency. First, we discuss the current situation and existing problems of health education in colleges and universities (CAUs). Second, we discuss how an AI-based algorithm and intelligent robot application are designed for HMR. Then, the proposed algorithm is optimized to improve the recognition rate. Third, we provide the specific experimental results. The final section discusses the results and the limitations. The research results promote the comprehensive application of intelligent robots and AI algorithms, contributing to the healthy development of college students.

## Enlightenment of Applying the AI Algorithm to the Development Path of Health Education

### Health Education in CAUs

**Figure 1:**
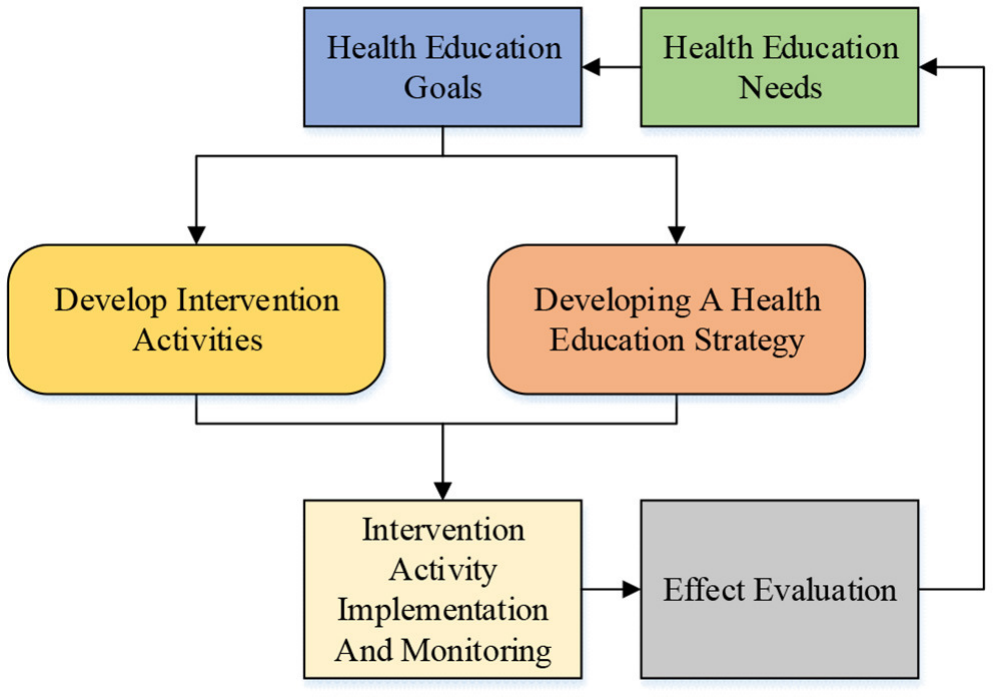
Path of college health education.

Physical education is an essential part of school health education. The school integrates sports and health promotion resources and holds sports activities to improve students' physique (Knijnik et al., 2019). Students' poor health awareness leads to short sports exercise time, relatively poor physique, and an increased obesity rate (García-Ceberino et al., 2020). Additionally, PE emphasizes students' sports skills and forms of education. Not paying attention to cultivating students' physique, exercise intensity, and exercise load deviate from school health education and is not conducive to cultivating students' individuality (García-Ceberino et al., 2019; Ilaria and Emanuela, 2019). Figure 1 shows the implementation mode of health education in domestic CAUs. The specific objectives are formulated by analyzing the needs of health education and getting corresponding feedback.

As shown in Figure 1, sports exercise is the main way students enhance their physique in CAUs. Sports exercise can promote the healthy growth of students and provide students with main learning motivation. Therefore, advanced AI algorithms are used in this work. The HMR algorithms for students during exercise needs to be studied and designed, which helps students carry out sports exercise strictly. It can also detect students' motion state and identity information in real-time to help students strictly manage their own health. This research breaks through the comprehensive application of the algorithm and provides students with more perfect management methods. Therefore, this research is a breakthrough.

### Development Path and Countermeasures of Health Education in CAUs

The following opinions are put forward to improve the teaching effect and management mode of school PE based on the understanding of the current situation of school health education:

1. The development of school health education depends on the setting of PE lessons. Hence, the resources based on school health should be integrated to expand health education lessons. PE should be the main body in the teaching process, supplemented by extracurricular sports activities. The training of students' sports skills should be valued during the lessons to improve students' physical fitness. Meanwhile, students should be taught to protect themselves against sports injury with emergency treatment skills. The elective courses should be combined with the teaching content of the compulsory lessons and integrate the sports health concepts. The school's medical department should initiate various health lectures and health consultation activities for students to help them understand the prevention and rehabilitation of common diseases (Arikan, 2020). Students' health education should also cross multiple disciplines to constantly improve the health education curriculum system.
2. Schools should initiate different sports activities to increase students' exercise time. For example, they can hold sports competitions, such as marathons, night running, sports meetings, and long-distance running. Sports competitions are developed to enhance students' enthusiasm to participate in sports exercise and improve their health awareness (Jeitler et al., 2020). Second, various student clubs should be launched. Students with the same hobbies should be organized to participate in different club activities. Schools should open up sports resources, such as fitness venues, physical examination centers, and swimming pools, to students more frequently.
3. Schools should value the management of PE lessons and extracurricular activities, increase PE courses and improve students' sports skills and physical fitness. Individual differences should be valued in teaching. Importantly, schools should ensure that students have sufficient after-school time for exercise and put forward requirements on students' exercise time and exercise frequency. The long-term follow-up health evaluations should be implemented to improve their health awareness and physical fitness. Besides, the schools should create an environment for sports exercise, establish a sports evaluation mechanism, increase the propaganda of sports exercise, and enhance students' enthusiasm for participating in sports.

**Figure 2:**
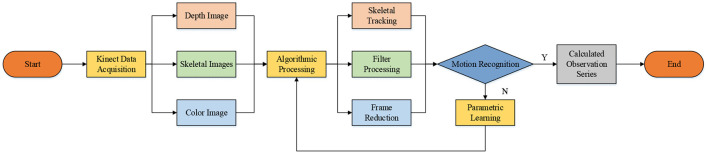
Design flowchart.

Combined with the goal of school health education, the HMR algorithm based on the Hidden Markov Model (HMM) is adopted. The HMM-based HMR algorithm can analyze and recognize the collected skeletal images of human motions and set the corresponding training program according to students' sports exercise needs. The purpose is to change students' living habits using science and technology. Figure 2 displays the algorithm process reported here. The process is mainly divided into the data acquisition layer, the Google algorithm processing layer, the operation recognition, and the application layer.

### Human Skeleton Feature Extraction Algorithm Using Kinect Sensor-Mounted Intelligent Robot

**Figure 3:**
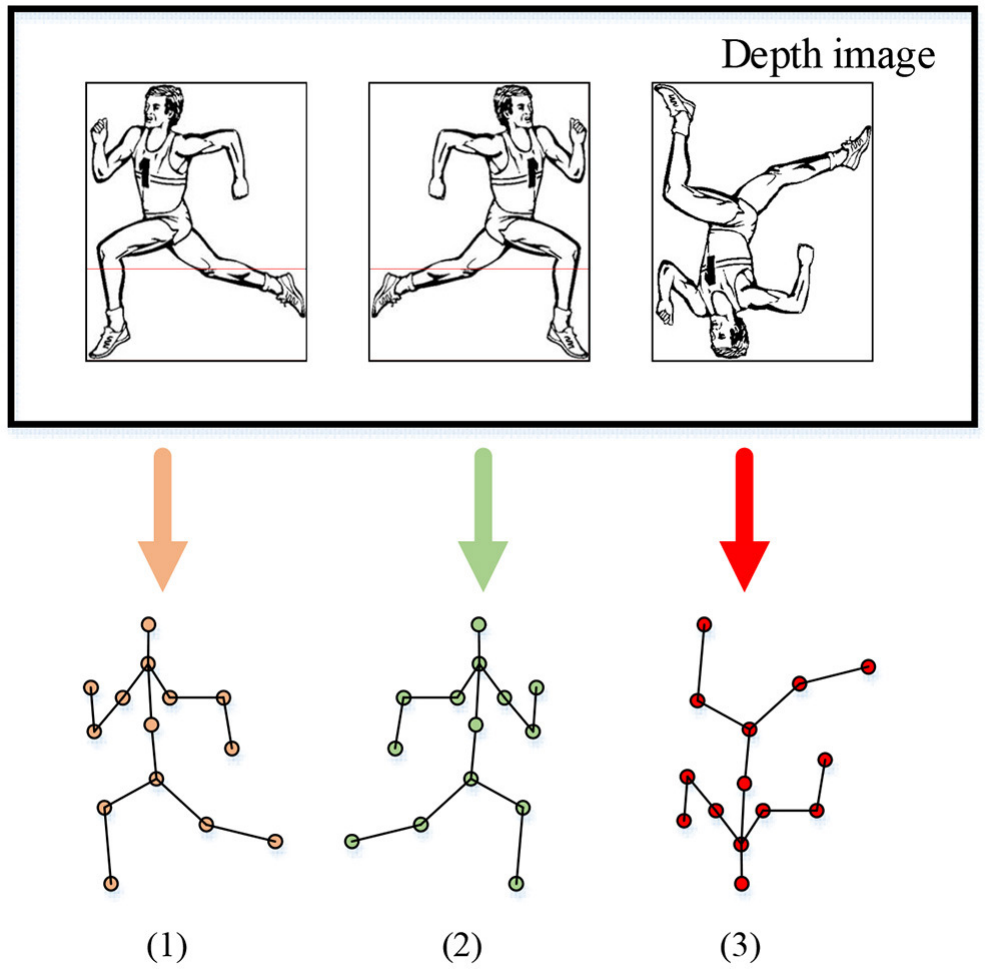
The data segmentation process of depth images.

The emergence of intelligent robots provides many promising technologies for human life. This study analyzes the recognition of sports exercise behavior of college students through AI algorithms and intelligent robotics technology. The manner how motions are represented affect the performance of the HMR algorithm. An excellent motion representation method can filter irrelevant information, streamline the data extraction, and describe the motion more accurately and comprehensively. The standard motion representation methods include human skeleton structuring and imaging method. The human skeleton structuring method regards the human body as a combination of multiple joints. In the recognition process, the joint position reflects the changes in human motions (Zhang et al., 2020). The imaging method extracts the spatial features of motions from the two-dimensional image by establishing a correlation model (Cao et al., 2019). Here, the Kinect sensor mounted on the intelligent robot collects images, including color images, infrared images, and depth images. In particular, the imaging principle of the depth image is to realize the visual reproduction process through reflection and capture. After the depth image is collected, the human joints are classified by eigenvalues to distinguish the human body from the depth image. The segmentation process of the depth image is drawn and shown in Figure 3.

The depth image changes when a person moves across the sensor field. Due to the difference between the pixel depth of the human body recognition and the background, the threshold method can classify the foreground and the background to obtain human body information (Keyvanpour et al., 2020). The threshold can be expressed as Equation (1).

(1) $$\begin{array}{l} f\left(x,y\right)=\{\begin{array}{cc} 1 & d\left(x,y\right)<\lambda \\ 0 & d\left(x,y\right)>\lambda \end{array} \end{array}$$

In Equation (1), *f*(*x, y*) indicates the logical judgment of the foreground pixel, 1 is the foreground pixel, and 0 is not the foreground pixel. Besides, *d*(*x, y*) represents the depth information of the pixel, λ denotes the threshold, which is related to the maximum depth *d*_max_, and the constant *K* is equal to 0.75. The threshold is calculated according to Equation (2).

(2) $$\begin{array}{l} \lambda =\left(d_{max}\cdot K\right) \end{array}$$

The threshold method can process the depth image, improve the accuracy of human foreground recognition, and facilitate the subsequent recognition of human skeleton information. Kinect can track and recognize the skeletons of two users simultaneously. It calculates the data of 25 human skeletal joint points, combines them to generate a skeleton diagram of the human body, and uses the skeletal joints to recognize human motions (Li et al., 2019a). Filtering algorithms can filter the collected depth images to improve the accuracy and precision of feature extraction. Thereby, they improve the accuracy of skeletal feature extraction from depth images and reduce skinny node data error (Liang et al., 2020). Selecting keyframes is the key to HMR. The keyframe technology can process the motion sequence and reduce the data dimension and the amount of data. The maximum reconstruction error and the number of keyframes are set as the optimization goals of keyframes. The frame reduction algorithm is used to obtain the final keyframe sequence to meet the reconstruction error and compression ratio requirements.

**Figure 4:**
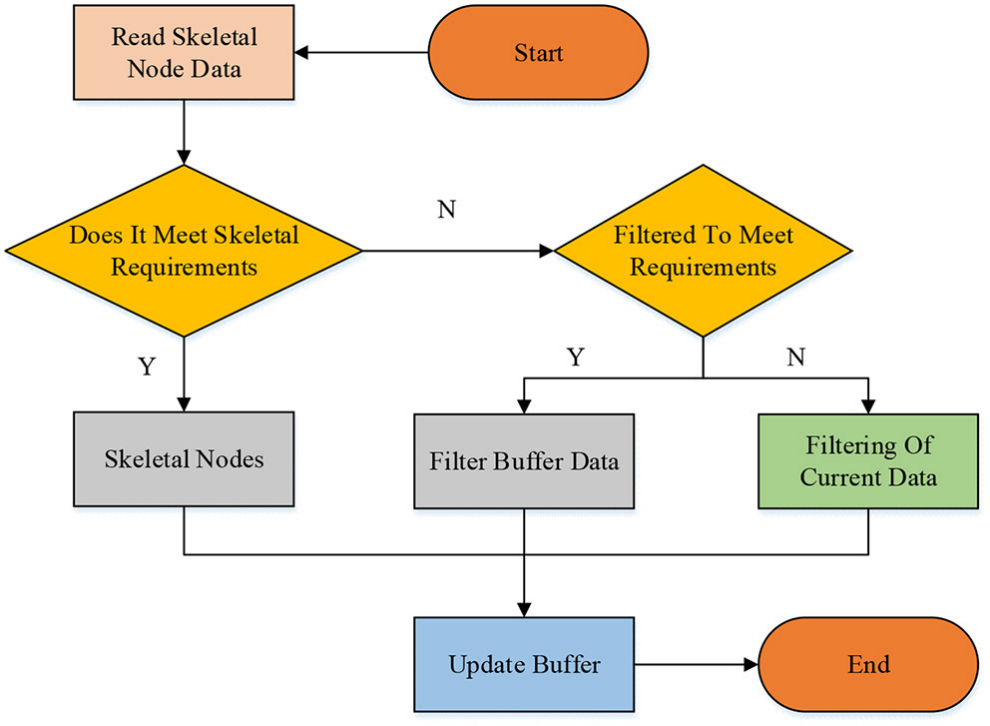
The limiting filtering processes.

Here, the amplitude-limiting filtering algorithm is used to process the data and eliminate the jitter of skeletal node data, the algorithm flow is shown in Figure 4. When the skeletal node data jitter exceeds the jitter radius threshold, it is considered that the skeletal recognition degree is low and needs to be corrected. The range of deviation radius threshold means that the data movement of skeletal nodes exceeds the jitter radius. When less than the jitter radius threshold, the node should be moved to eliminate the impact of skeletal jitter.

After filtering, the spikes and noise interference of the data are removed, and smoother skeletal node data are obtained. Different motions, such as pull-ups and sit-ups, are analyzed and decomposed. According to the change process of the motion, skeletal nodes at various vital positions are selected as features to convert human motion into motion sequences. Therefore, the human skeleton data captured by the Kinect sensor undergo feature separation, skeletal tracking, and filtering operations to obtain feature information of skeletal joints in human motion. Then these feature data are utilized for training HMM to get HMR results for different movements.

## Design of Sports Exercise Model Under HMM

### Student Exercise Behavior-Oriented HMR Algorithm Based on HMM

**Figure 5:**
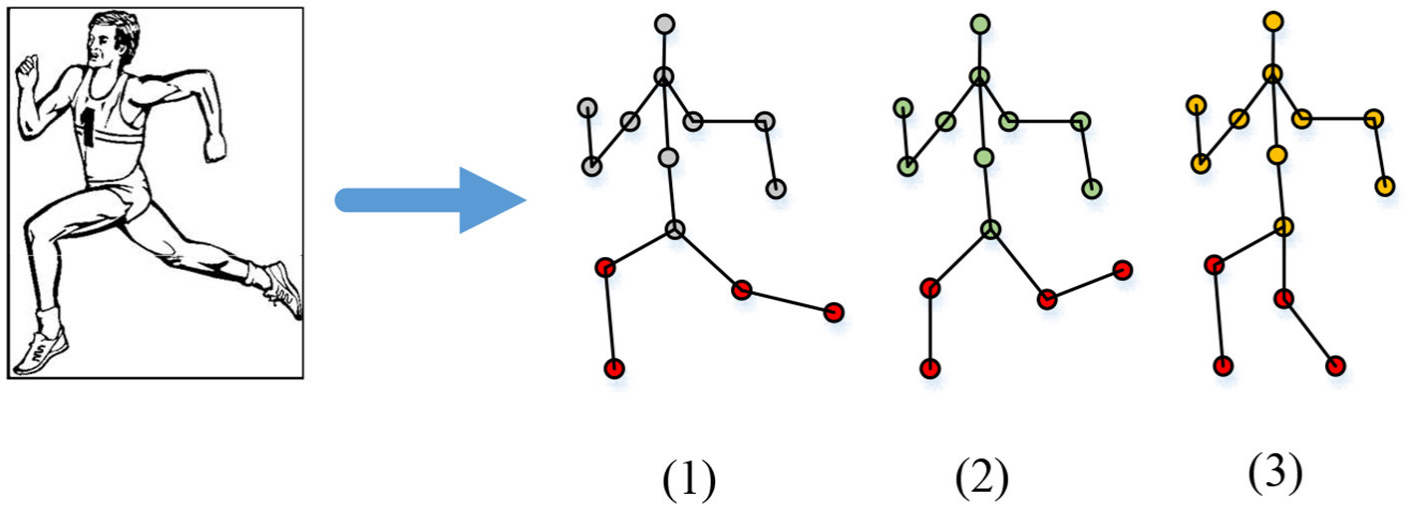
Motion representation method based on the human body structure.

Human motion is a time-varying motion sequence. A motion may contain dozens or hundreds of frame sequences; thus, recognizing human motion is very complicated (Gurbuz and Amin, 2019). Generally, HMR extracts the features of the collected human motion data and describes human motion through the features. Then, the classifier is used for learning and training to recognize the motions. Here, the method based on the human body structure expresses the human motion information. The three-dimensional coordinates of the skeletal joint points are used to represent the human motion state. The human skeleton node is established as the basis of motion recognition. The HMM is used to classify the motions (Jaouedi et al., 2020). Figure 5 shows the motion representation method based on the human body structure.

The HMM describes a Markov process with unknown parameters. It is a statistical probability model of a double random process, a dynamic Bayesian network with a simple structure. The HMM is often used in speech, pattern, and motion recognition (Shi et al., 2020). A Markov chain is a discrete random process with Markov properties. An arbitrary state sequence *X*_*n*_ is in the state of θ_1_, θ_2_, …, θ_*n*_ within a particular period and in the state of *m* at time *q*_*m*_. The state of the next unit moment is only related to the last moment, as shown in Equation (3).

(3) $$\begin{array}{l} P(X_{m+1}=q_{m+1}|X_{m}=q_{m},X_{m-1}=q_{m-1},\ldots ,X_{1}=q_{1})\\ \;=P\left(X_{m+1}=q_{m+1}|X_{m}=q_{m}\right) \end{array}$$

In Equation (3), *q*_1_, *q*_2_, …, *q*_*m*+1_ belongs to θ_1_, θ_2_, …, θ_*n*_; *X*_*n*_ is a Markov chain.

**Figure 6:**
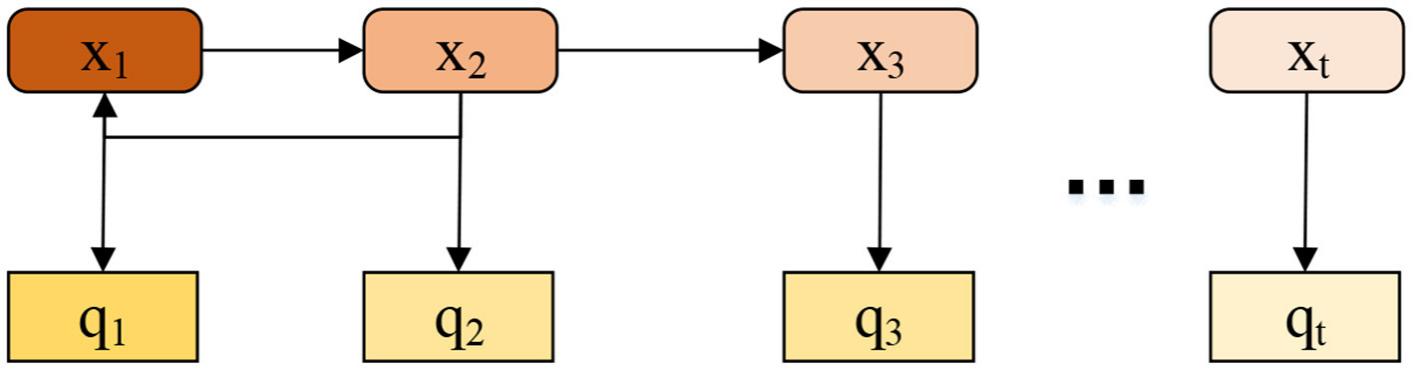
A schematic diagram of HMM.

In the process of HMR, motion sequences meet HMM's requirements (Figure 6) under a certain probability. Markov chain describes the alignment process of standard sequence and motion sequence. Multiple random methods can be characterized by conversion under a certain probability (Gao et al., 2020).

The HMM divides the human motion process into many continuous state sequences and takes each motion in the state space as a state of the motion sequence. Each state can only be linked by probability. Therefore, the probability relationship of transferring from one state to another can be used to describe human motions (Hu and Lee, 2020). The HMM regards human behavior as a real-time multi-state transformation process and observes the hidden state by sensing the feature vector generated in the event. Accordingly, it identifies the specific motion state of the human body.

In HMM, the motion state cannot be observed directly. However, the vectors can express various states under the probability density distribution, and the active state can be obtained by observing vectors. The model contains the following two stochastic processes: (1) The hidden Markov chain with a certain number of states and (2) observed values corresponding to the state in a specific state.

The HMM can be described by two state sets and three probability matrices.

1. Hidden states refer to the states implied in the Markov model. These states have Markov properties, where θ_1_, θ_2_, …, θ_*n*_ represents the definition of these hidden states, *N* stands for the number of hidden states, and *q*_*t*_ refers to the state represented by the Markov chain at time *t* (Mottaghi et al., 2020).
2. The observable state refers to the state connected to the hidden condition that can be directly observed. Here, *V*_1_, *V*_2_, …, *V*_*m*_ represents the obtained *M* observations, *M* refers to the number of observations in a particular state, and *O*_*t*_ represents the observations at time *t*.
3. The initial state probability matrix refers to the probability matrix of the hidden state in the initial state *t* = 1, which is expressed by the probability vector of the initial state as follows:
  (4) $$\begin{array}{l} \pi =\left(\pi_{1},\pi_{2},\ldots ,\pi_{n}\right) \end{array}$$
  (5) $$\begin{array}{l} \pi_{i}=P\left(q_{i}=\theta_{i}\right),1\leq i\leq N \end{array}$$
4. The implicit state transition probability matrix refers to the probability of transition between different states in HMM, which can be expressed as follows:
  (6) $$\begin{array}{l} A={\left(a_{ij}\right)}_{N^{*}N} \end{array}$$
  (7) $$\begin{array}{l} a_{ij}=P\left(q_{t+1}=\theta_{j}|q_{t}=\theta_{i}\right) \end{array}$$
  In Equations (6) and (7), *a*_*ij*_ represents the probability of state θ_*j*_ at time *t* + 1 under the premise of state θ_*i*_ at time *t*.
5. The observation state transition probability matrix refers to the probability that the observation state is *O*_*t*_ when the hidden state is θ_*j*_ at time *t*, and the probability matrix of the observation value is represented by *B*, which can be defined as follows:
  (8) $$\begin{array}{l} B={\left(b_{jk}\right)}_{N^{*}M} \end{array}$$
  (9) $$\begin{array}{l} b_{jk}=P\left(O_{t}=V_{k}|q_{t}=\theta_{j}\right),1\leq i\leq N,1\leq k\leq M \end{array}$$
  Therefore, the HMM contains two parts: the Markov process and the random process. The state sequence is output in the Markov process, and the observation value sequence and the state sequence correspond to the production in the random process. Hence, the HMM can be expressed as Equation (10).
  (10) $$\begin{array}{l} \lambda =\left(\pi ,A,B\right) \end{array}$$

### Algorithm Optimization of HMM

**Figure 7:**
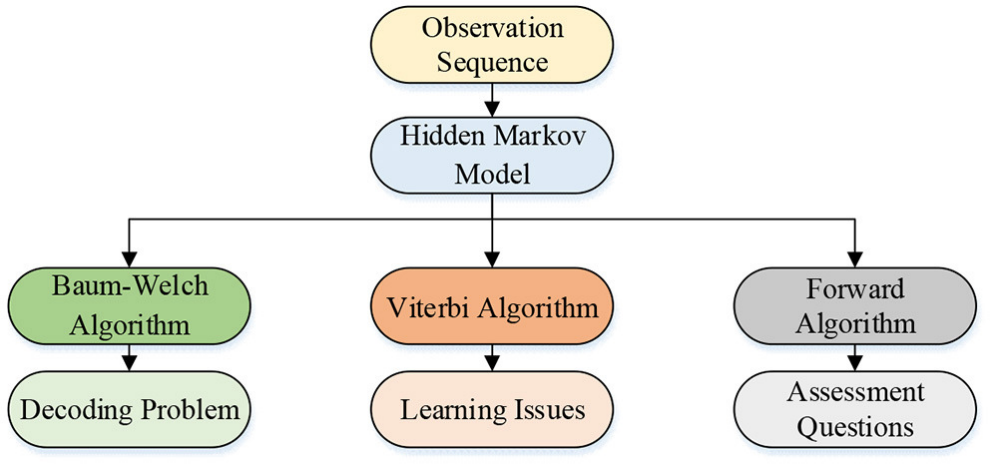
HMM algorithm relationship.

The fundamental problems of HMM include evaluation problems, decoding problems, and learning problems (Li et al., 2020a). Corresponding algorithms are proposed for these problems. Figure 7 reveals the algorithm relationship.

1. The evaluation problem refers to calculating the maximum probability *P*(*O*|λ) of the observation sequence *O* under λ for a given HMM λ = (π, *A, B*) and observation sequence *O*. All possible hidden sequences can be obtained through the exhaustive method. The possibility of the hidden sequence generating the observed sequence is calculated. However, this method has high time complexity. Suppose the model parameters are known, and the occurrence probability of the observation sequence is given. In that case, the maximum probability of the observer sequence is found through the forward–backward algorithm to obtain the HMM (Miki et al., 2020). The forward–backward algorithm calculates the probability *P*(*O*|λ) of the observation sequence. For a given HMM λ = (π, *A, B*), the algorithm is used to obtain the probability *P*(*O*|λ) of the observation sequence *O*_1_, *O*_2_, …, *O*_*t*_.
  Forward algorithm: the forward probability is defined first. For a given model λ = (π, *A, B*), the probability is defined until time *t*, the observation sequence is *O*_1_, *O*_2_, …, *O*_*t*_, and the state is *q*_*i*_. The equation is expressed as follows:
  (11) $$\begin{array}{l} a_{t}\left(i\right)=P\left(O_{1},O_{2},\ldots ,O_{T},P=\theta_{i}|\lambda \right),2\leq t\leq T \end{array}$$
  The forward probability *a*_*t*_(*i*) and the observation sequence probability *P*(*O*|*a*) can be obtained through recurrence and initialized as expressed in the following:
  (12) $$\begin{array}{l} a_{i}\left(i\right)=\pi_{i}b_{i}\left(O_{i}\right),1\leq i\leq N \end{array}$$
  The forward probability defines the observation sequence so far and the current state. Therefore, the equation contains the initial state and recent observation probability (Li et al., 2019b).
  Recurrence:
  (13) $$\begin{array}{l} a_{t+1}=\left[\sum_{i=1}^{N}a_{t}\left(i\right)a_{ij}\right]b_{j}\left(O_{t+1}\right),1\leq t\leq T\\ \;-1,1\leq j\leq N \end{array}$$
  Termination:
  (14) $$\begin{array}{l} P\left(O|\lambda \right)=\sum_{i=1}^{N}a_{T}\left(i\right) \end{array}$$
  (15) $$\begin{array}{l} b_{j}\left(O_{t+1}\right)=b_{jk}|O_{t+1}=V_{k} \end{array}$$
  The local calculation can improve the computational efficiency of the forward algorithm. Based on the state sequence combined with the path structure, the forward probability has recurred to the global, obtaining the probability.
  The backward probability is defined in the backward algorithm as the probability of a given model λ = (π, *A, B*), when the state at time *t* is *q*_*i*_, the partial observation sequence from *t* + 1 to *T* is *O*_*i*+1_, *O*_*i*+2_, …, *O*_*T*_, and the equation is:
  (16) $$\begin{array}{l} \beta_{t}\left(i\right)=P\left(O_{i+1},O_{i+2},\ldots ,O_{T}|q_{t}=\theta_{i},\lambda \right) \end{array}$$
  Initialization:
  (17) $$\begin{array}{l} \beta_{T}\left(i\right),1\leq i\leq N \end{array}$$
  Recurrence:
  (18) $$\begin{array}{l} \beta_{t}\left(i\right)=\sum_{j=1}^{N}a_{ij}b_{j}\left(O_{t+1}\right)\beta_{t+1}\left(j\right) \end{array}$$
  (19) $$\begin{array}{l} t=T-1,T-1,\ldots ,1 \end{array}$$
  (20) $$\begin{array}{l} 1\leq j\leq N \end{array}$$
  Termination:
  (21) $$\begin{array}{l} P\left(O|\lambda \right)=\sum_{i=1}^{N}\beta_{t}\left(i\right) \end{array}$$
  By calculating the transition probability of *N* states *q*_*j*_ at the time *t* + 1 and the observation probability of observation *O*_*t*+1_ in this state, the backward probability of the observation sequence after *q*_*j*_ is calculated (Liu et al., 2019).
2. The learning problem is to estimate the parameter λ of the model for a given observation sequence *O* to maximize the probability *P*(*O*|λ) of the observation sequence under λ. The problem is to derive the model parameters from the observation sequence and estimate the parameters using the maximum likelihood estimation method. It can be calculated using the Baum–Welch algorithm (Li et al., 2020b). The algorithm flow of the Baum–Welch algorithm is discussed as follows: Initializing model parameters, selecting $a_{ij}^{0},\beta_{j}^{0},\pi_{i}^{0}$, obtaining model λ^0^ = (π^0^, *A*^0^, *B*^0^), and solving the two intermediate variables γ_*t*_(*i*) and ξ_*t*_(*i,j*); γ_*t*_(*i*) represents the model λ and the observation sequence *O*, the probability that the hidden state is *q*_*i*_ at time *t*. The equation is expressed as follows:
  (22) $$\begin{array}{l} \gamma_{t}\left(i\right)=P\left(\theta_{t}=q_{i}|O,\lambda \right) \end{array}$$
  In Equation (22), ξ_*t*_(*i, j*) represents the probability of model λ and observation sequence *O*, the hidden state at time *t* is *q*_*i*_, and the hidden state at time *t* + 1 is *q*_*j*_. The equation is expressed as follows:
  (23) $$\begin{array}{l} \xi_{t}\left(i,j\right)=P\left(\theta_{t}=q_{i},\theta_{t+1}=q_{j}|O,\lambda \right) \end{array}$$
  According to the definitions of forwarding probability and backward probability, intermediate variables γ_*t*_(*i*) and ξ_*t*_(*i, j*) can be written as:
  (24) $$\begin{array}{l} \gamma_{t}\left(i\right)=\frac{a_{t}\left(i\right)\beta_{t}\left(i\right)}{\sum_{j=1}^{m}a_{t}\left(i\right)\beta_{t}\left(i\right)} \end{array}$$
  (25) $$\begin{array}{l} \xi_{t}\left(i,j\right)=\frac{a_{t}\left(i\right)a_{ij}b_{j}\left(O_{t+1}\right)\beta_{t+1}\left(j\right)}{\sum_{p=1}^{m}\sum_{q=1}^{m}a_{t}\left(p\right)a_{pq}b_{q}\left(O_{t+1}\right)\beta_{t+1}\left(q\right)} \end{array}$$
  The two intermediate variables are calculated according to the following expressions:
  (26) $$\begin{array}{l} a_{ij}=\frac{\sum_{t=1}^{T}\xi_{t}\left(i,j\right)}{\sum_{t=1}^{T}\gamma_{t}\left(i\right)} \end{array}$$
  (27) $$\begin{array}{l} b_{j}\left(k\right)=\frac{\sum_{t=1}^{T}\gamma_{t}\left(j\right)I\left(O_{t}=V_{k}\right)}{\sum_{t=1}^{T}\gamma_{t}\left(j\right)} \end{array}$$
  (28) $$\begin{array}{l} \pi_{i}=\gamma_{1}\left(i\right) \end{array}$$
  Equation (29) indicates *I* of Equation (27).
  (29) $$\begin{array}{l} I=\{\begin{array}{cc} 1 & O_{t}=V_{k}\\ 0 & O_{t}\neq V_{k} \end{array} \end{array}$$
  The results obtained through the convergence process are used as the model's parameters.
3. The decoding problem is to calculate the implicit sequence *X* that is most likely to produce the observation sequence *O* for a given model λ = (π, *A, B*) and observation sequence *O*, and maximize the probability *P*(*X*|*O*, λ) (Vu and Kim, 2018a). The Viterbi algorithm can be used to find the optimal path of probability. First, δ(*i*) is defined as the maximum probability of the observation value *O*_1_, *O*_2_, …, *O*_*t*_ obtained when the state is *q*_1_, *q*_2_, …, *q*_*t*_ at time *t* when *q*_*t*_ = θ_*i*_. The algorithm flow is provided as follows:
  Initialization:
  (30) $$\begin{array}{l} \delta_{1}\left(i\right)=\pi_{i}b_{i}\left(O_{1}\right),1\leq i\leq N \end{array}$$
  (31) $$\begin{array}{l} \varphi_{1}\left(i\right)=0,1\leq i\leq N \end{array}$$
  Recurrence:
  (32) $$\begin{array}{l} \delta_{t}=\underset{1\leq i\leq N}{max}\left[\delta_{t-1}\left(i\right)a_{ij}\right],2\leq t\leq T,1\leq j\leq N \end{array}$$
  (33) $$\begin{array}{l} \varphi_{t}\left(j\right)=\underset{1\leq i\leq N}{argmax}\left[\delta_{t-1}\left(i\right)a_{ij}\right],2\leq t\leq T,1\leq j\leq N \end{array}$$
  Termination:
  (34) $$\begin{array}{l} P^{*}=\underset{1\leq i\leq N}{max}\left[\delta_{T}\left(i\right)\right] \end{array}$$
  (35) $$\begin{array}{l} q_{T}^{*}=\underset{1\leq i\leq N}{argmax}\left[\delta_{T}\left(i\right)\right] \end{array}$$
  Optimal path backtracking:
  (36) $$\begin{array}{l} q_{T}^{*}=\varphi_{t+1}\left(q_{t+1}^{*}\right),t=T-1,T-2,\ldots ,1 \end{array}$$

In Equations (30–36), $Q^{*}=q_{1}^{*},q_{2}^{*},\ldots ,q_{T}^{*}$ represents the sequence in a specific state when the probability *P*(*Q, O*|λ) is maximized by the given observation sequence *O*_1_, *O*_2_, …, *O*_*T*_ and model λ = (π, *A, B*). The optimal path can find according to the dynamic programming method. If the route passes $q_{t}^{*}$ at time *t*, the partial path from $q_{t}^{*}$ to $q_{T}^{*}$ of the path is optimal for all paths.

The core of using HMM for HMR is to determine the hidden state. The quality of the initial model affects the training results and the accuracy of HMR. Therefore, the motion sequence corresponding to the human body motion process is averaged into the segment *N*, corresponding to the *N* state in the model. All motion sequences in the motion recognition generate *N* state transition sequences. These state transition sequences are used to calculate the initialization parameters of each motion. In addition, $N_{i,j}^{m}=N^{m}\left(s_{t+1}=q_{j}|s_{i}=q_{i}\right)$ denotes the number of states *q*_*i*_ in the *t*th frame and the number of states *q*_*j*_ in the *t* + 1-th frame in the *m*th state sequence, and $N_{i}^{m}=N^{m}\left(s_{1}=q_{i}\right)$ indicates the number of states *q*_*i*_ in the first frame in the *m*th state sequence. Therefore, the equation in the initial state probability $\pi_{i}^{0}$ is expressed as follows:

(37) $$\begin{array}{l} \pi_{i}^{0}=P\left(s_{1}=q_{i}\right)=\frac{\sum_{m}N_{i}^{m}}{\sum_{i}\sum_{m}N_{i}^{m}} \end{array}$$

Equation (38) expresses the probability that the first frame's state of this motion is *q*_*i*_ and calculates the number of state transitions for all state sequences to find the adjacent frame. The transition probability *a*_*i, j*_ of the initial state is expressed as follows:

(38) $$\begin{array}{l} a_{i,j}=P\left(s_{t+1}=q_{j}|s_{i}=q_{i}\right)=\frac{\sum_{m}N_{i}^{m}}{\sum_{i}\sum_{m}N_{i}^{m}} \end{array}$$

### Introduction to Algorithm Identification Path and Data Set

**Figure 8:**
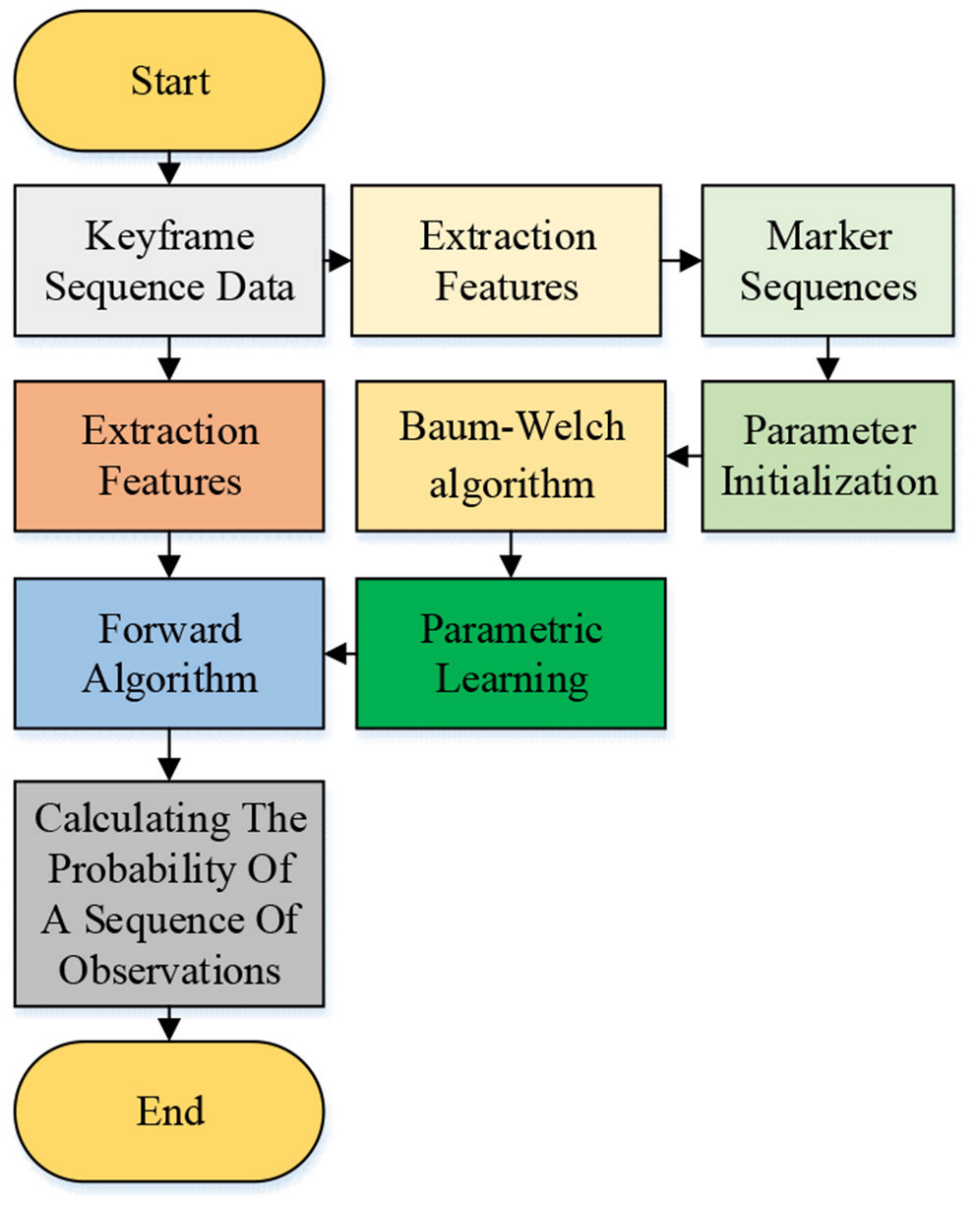
Model training and recognition process.

This study uses the Baum–Welch algorithm to learn the model parameters. The initialized parameter λ^0^ = (π^0^, *A*^0^, *B*^0^) is input into the model for parameter learning to obtain the parameter λ^*i*^ = (π^*i*^, *A*^*i*^, *B*^*i*^) of each motion. Then, the forward algorithm is performed on these motions to obtain the probability *P*(*O*|λ^*i*^). The motion with the largest *P*(*O*|λ^*i*^) is taken as the recognition result of the test motion (Ding et al., 2019). Figure 8 illustrates the model's training and recognition process.

ChaLearn Gesture Data 2013 (CGD2013) and Hochschule Der Medien 05 (HDM05) data sets are selected. They are used to train and test the proposed HMM-based HMR algorithm on Matlab R2014a to verify the recognition performance of motion states. The CGD2013 data set contains 11,000 color images of 20 kinds of gestures (550 pictures for each gesture). This experiment selects 400 images from each type of gesture picture, a total of 8,000 images as the training data set of static gesture recognition; the remaining 3,000 photos are used as the test data set. The HDM05 data set contains motion capture data in 3D revolution (C3d) and advanced streaming format/ad Muncher coding (ASF/AMC) data formats with more than 3 h of system and detailed records. In addition, HDM05 contains more than 70 motion categories performed by different actors.

## Results and Discussion

### Basic Situation of Motion Recognition

**Figure 9:**
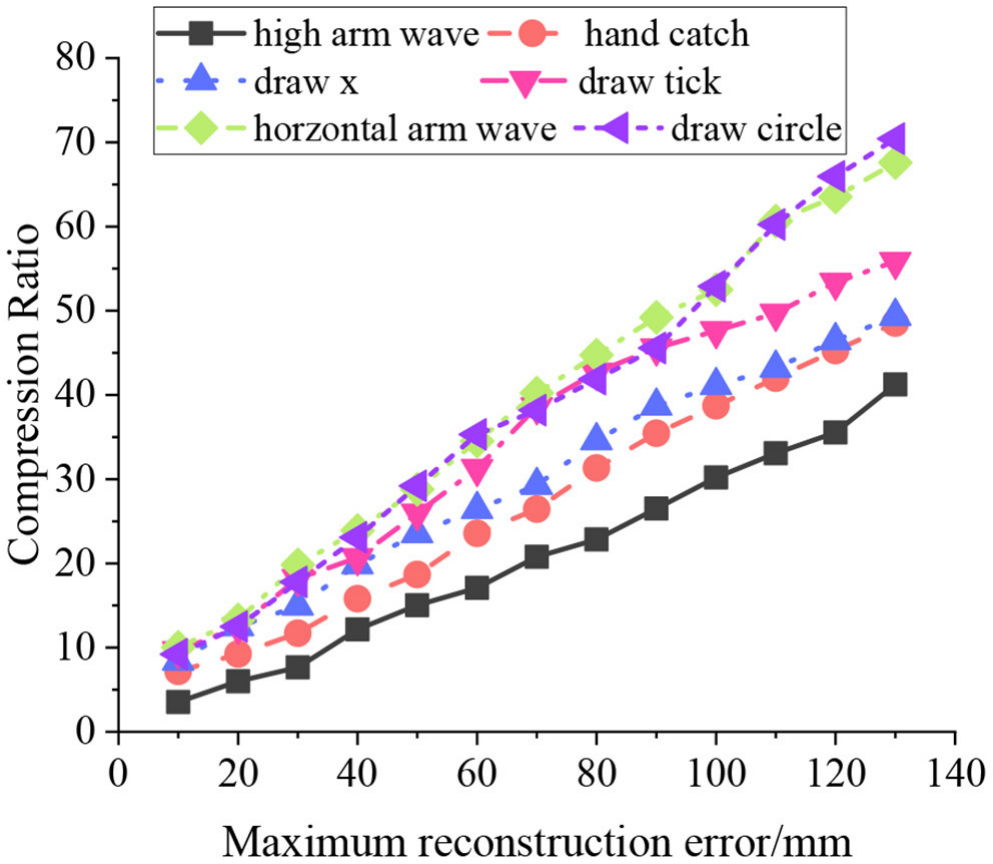
Model training and recognition results.

By applying the AI algorithm and Intelligent robotics technology, this study analyzes the characteristics of college students' sports behavior and the path of health education. Notably, the Kinect senor-mounted intelligent robot can analyze the skeleton structure of college students during sports exercises and extract the features. Also, it helps to point out the shortcomings of students' sports exercise and provides them with better sports exercise methods. Therefore, the research content can promote students' sports exercise and the effect of college PE. The various motions in the HDM05 database are used to verify the image processing performance of the frame subtraction algorithm and filtering algorithm through motion identification. The test results are shown in Figure 9.

As in Figure 9, the recognition of different motions by the algorithm presents a linear relationship. For example, the recognition rate for “high arm waves” motion is the worst, about 40% at best. The recognition rate for “draw circle” motion is the best, about 75%. The maximum reconstruction error is linearly related to the motion compression ratio. The compression ratio increases with the maximum reconstruction error increase, independent of the motion type. When the maximum reconstruction error is <10 mm, the compression ratio changes between 5 and 10. Presumably, the compression ratio of different motion sequences increases under the same maximum error and slow-motion trend. For violent motion sequences, the compression ratio needs to be reduced.

### Model Training Analysis

**Figure 10:**
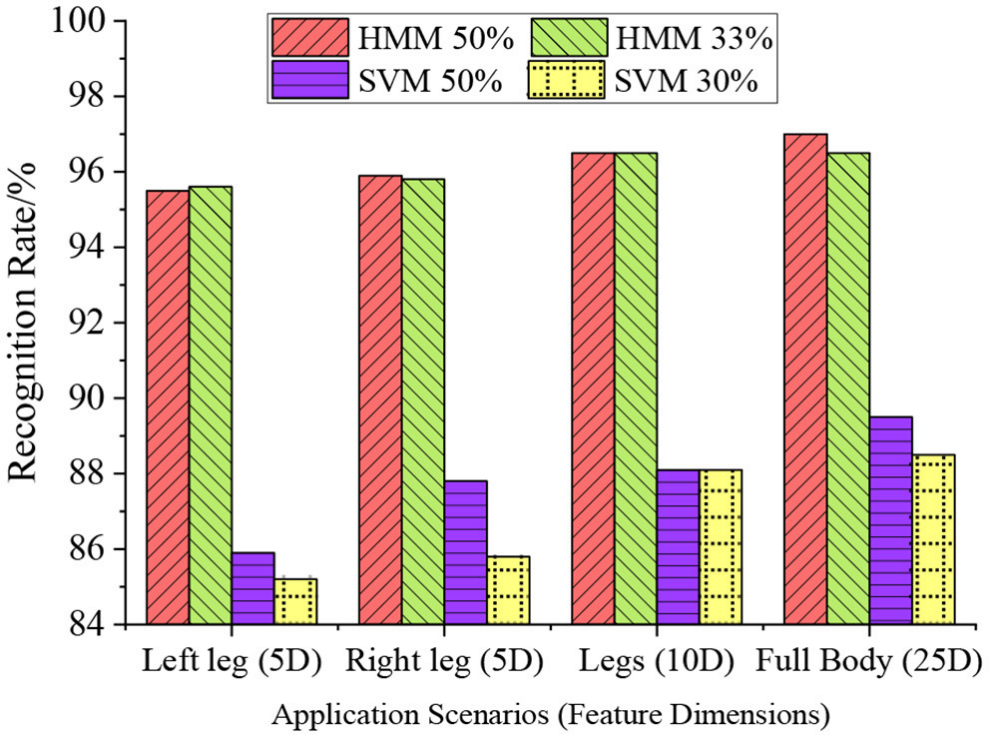
Comparison of the recognition rates of HMM and SVM algorithms when the number of training samples is 50 and 33%.

The different numbers of training samples are used to compare the recognition performance of HMM and SVM algorithms, thereby verifying the performance of the designed algorithm for HMR. The number of samples set is 50 and 33% of the total number of samples (number of training samples/total number of samples). The results are shown in Figure 10.

As shown in Figure 10, the number of training samples has little effect on the recognition performance of the HMM. When the number of training samples is reduced from 50 to 33%, the reduction of recognition rate is no more than 1.5%. The recognition effect of SVM is greatly affected by the number of training samples. At the same time, increasing the number of human motion features can improve the recognition rate of the algorithm, reaching 97%. With the increase of the number of features, the recognition rate of SVM decreases to 85.2%.

### Recognition Performance Analysis

**Figure 11:**
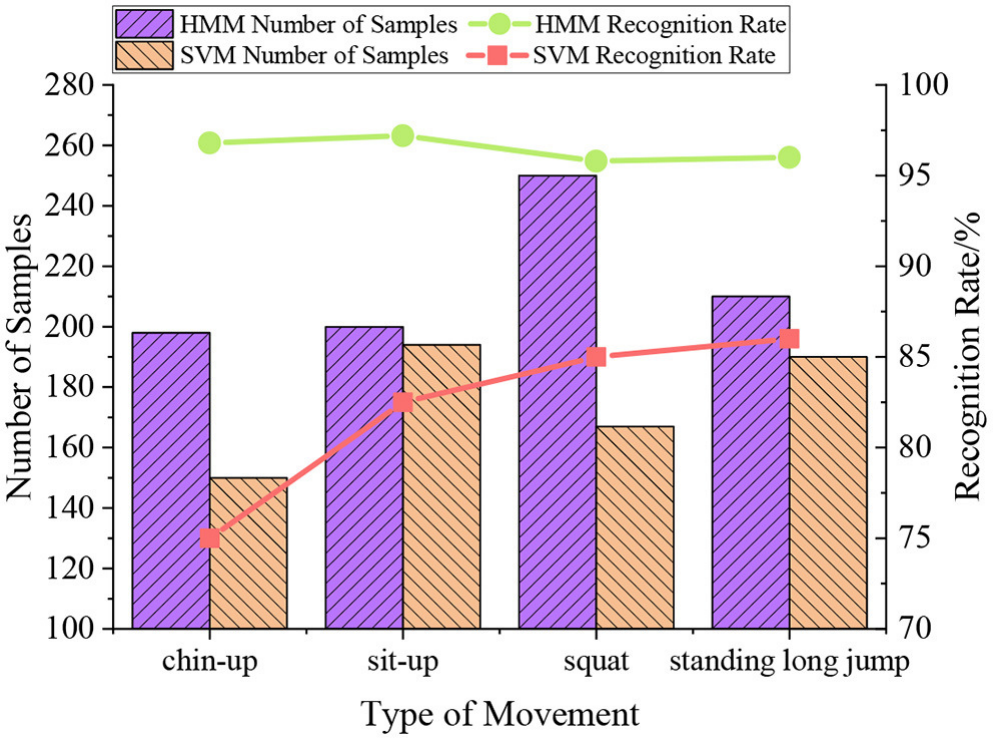
Model training and recognition process.

A recognition test is performed on different motion types with varying numbers of samples to test the recognition performance of the motion recognition algorithm for various motions. The results are shown in Figure 11.

In Figure 11, the HMM algorithm obtains better recognition results than the SVM algorithm. The recognition rate of the HMM algorithm is more than 95%, while the recognition rate of the SVM algorithm is between 75 and 85%. The recognition rate of the algorithm designed here is high. However, there are differences in the recognition rate of different motion types. The reason may be that the motion time complexity of varying motion types leads to different recognition effects of the algorithm. Still, the recognition rate of the overall algorithm is more than 95%. The recognition rate of the SVM algorithm for different motion types is far lower than that of the algorithm reported here.

### Performance Comparison of Different Algorithms

**Figure 12:**
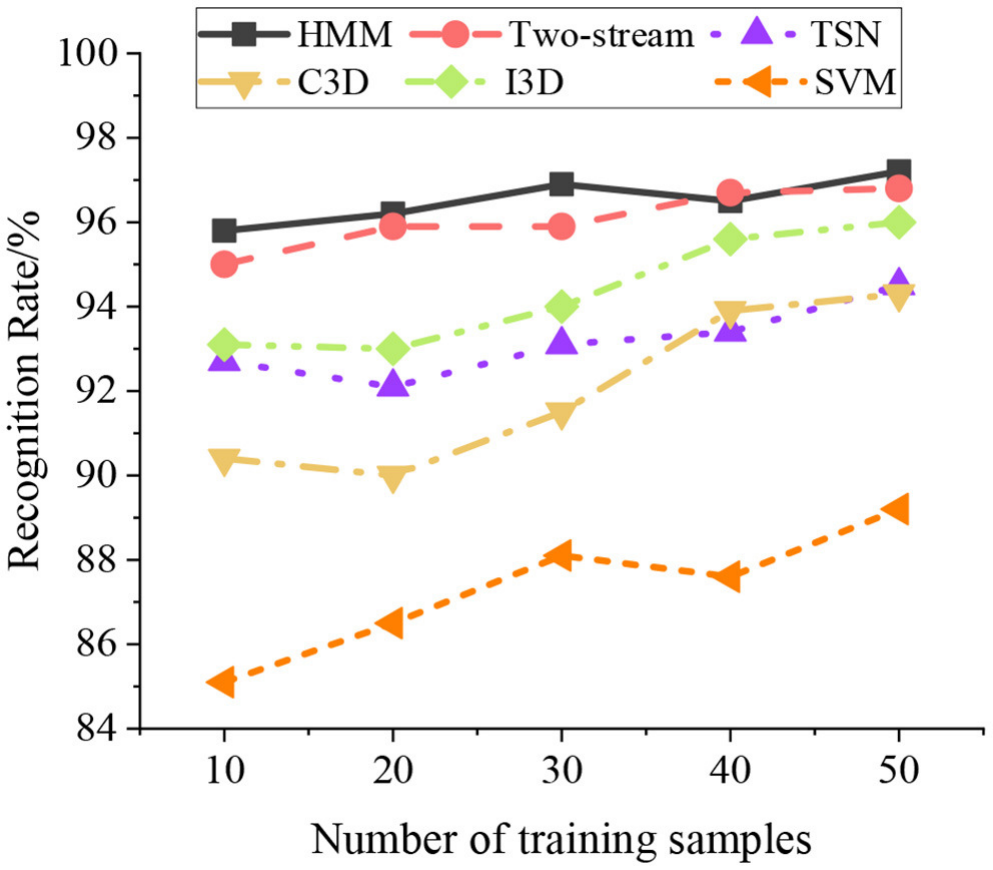
Model training and recognition process.

The HMM algorithm is compared with several mainstream HMR algorithms to verify its effectiveness, including time-sensitive network (TSN) (Ding et al., 2018), 3D convolution (C3D) (Zhou et al., 2017), two-stream inflated 3D ConvNet (I3D) (Vu and Kim, 2018b), and support vector machines (SVMs). Figure 12 provides the performance comparison results.

Figure 12 shows that the HMM algorithm has a higher recognition rate than other algorithms and can achieve the same recognition effect as I3D and TSN. The overall recognition rate of HMM algorithm is more than 96%, which shows an excellent effect in HMR. On comparison, SVM presents the worst recognition effect, and the overall recognition rate is between 85 and 87%. Additionally, HMM algorithm is weakly dependent on the number of samples. In other words, HMM only needs a small number of samples to get a high recognition rate.

### Discussion

This study aims to use science and technological approaches to help students cultivate healthy living habits and establish a reasonable model for college health education. The HMR algorithm is designed based on AI and intelligent robotics technologies. Specifically, the Kinect sensor-mounted intelligent robot can identify students' sports exercise motions. The Kinect-captured data provide reliable sports performance data for teachers and help formulate strategies conducive to students' health education. Finally, the proposed (AI + intelligent robot)-enabled HMM-based HMR algorithm is tested. The experiments show that the proposed HMM-based HMR algorithm can effectively recognize motions, with an accuracy reaching over 96%. Additionally, the proposed HMM-based HMR algorithm has low sensitivity to the number of samples. It can achieve a high recognition rate given only a few samples. Moreover, compared with other literature algorithms, HMM has a better recognition effect and can meet the actual needs of motion recognition. The HMM-based HMR algorithm recognizes motions by analyzing the collected human skeleton images. According to students' sports exercise needs, the proposed algorithm can effectively formulate related training programs to identify students' behavior characteristics in sports exercise.

## Conclusion

Using the AI algorithm and intelligent robotic technology, this study analyzes the problems in school health education to identify students' exercise motions. It also suggests improved exercising strategies for students. First, the problems in school health education are discussed, and the corresponding solutions are determined. Then, HMM is used to process the collected students' movement depth images, identify students' behavior, and extract the behavior features. The experimental results show that the recognition rate of the proposed HMM-based HMR algorithm is more than 96%, so it outmatches other behavior recognition algorithms. Additionally, HMM is less dependent on the number of samples. Therefore, HMM can identify students' sports status and identity information on a large scale, provide students with more advantageous sports exercise methods, and simultaneously analyze students' sports status. The research content provides an important reference for establishing a scientific and reasonable path of health education in CAUs and contributes to improving teaching quality in CAUs.

However, there are still some deficiencies. Due to the limited accuracy of the Kinect sensor, the model has insufficient detection ability for human skeletal nodes. Therefore, it cannot accurately identify the hand's motion state and detect its standardization when the subject performs a pull-up. Besides, only data sets are used to test the algorithm, ignoring the influence of complex factors in the actual application environment. Therefore, the subsequent research will use higher precision sensors for experiments and continuously optimize the recognition ability of the algorithm through practical tests.

## Data Availability Statement

The raw data supporting the conclusions of this article will be made available by the authors, without undue reservation.

## Ethics Statement

The studies involving human participants were reviewed and approved by Chongqing University Ethics Committee. The patients/participants provided their written informed consent to participate in this study. Written informed consent was obtained from the individual(s) for the publication of any potentially identifiable images or data included in this article.

## Author Contributions

Both authors listed have made a substantial, direct, and intellectual contribution to the work and approved it for publication.

## Conflict of Interest

The authors declare that the research was conducted in the absence of any commercial or financial relationships that could be construed as a potential conflict of interest.

## Publisher's Note

All claims expressed in this article are solely those of the authors and do not necessarily represent those of their affiliated organizations, or those of the publisher, the editors and the reviewers. Any product that may be evaluated in this article, or claim that may be made by its manufacturer, is not guaranteed or endorsed by the publisher.
